# The relationship between pelvic floor functions and vaginal microbiota in 6–8 weeks postpartum women

**DOI:** 10.3389/fmicb.2022.975406

**Published:** 2022-11-03

**Authors:** Yakun Zhang, He Yang, Li Lin, Wenlan Yang, Guangwu Xiong, Guolan Gao

**Affiliations:** ^1^Savaid Medical School, University of Chinese Academy of Sciences, Beijing, China; ^2^Department of Obstetrics and Gynecology, Peking University International Hospital, Beijing, China

**Keywords:** pelvic floor function, vaginal microbiota, 16S rRNA, postpartum women, relationship research

## Abstract

The impairment of pelvic floor muscle functions and *Lactobacillus*-deficient vaginal microbiota is common in postpartum women. However, few studies have explored the correlation between pelvic floor muscle functions and vaginal microbiota. Given this research gap, our study aims to investigate any potential association between these two conditions of postpartum women (6–8 weeks after childbirth). A total of 230 women who required postpartum pelvic floor function examination at Peking University International Hospital from December 2021 to April 2022 were enrolled in this study. The collected questionnaire information included progestational weight, body mass index (BMI), weight gain during pregnancy, neonatal weight, delivery type, multiparity, postpartum time, and urinary incontinence (UI). A total of 187 samples of vaginal secretions were collected, and the vaginal microbiota was detected by 16S rRNA sequence analysis. Finally, 183 samples were analyzed in the trial. All individuals were divided into two groups according to the results of pelvic floor muscle assessment to explore the difference between the incidence of postpartum urinary incontinence and vaginal microbiota. We found that the prevalence of UI was higher in the group with weakened pelvic floor muscles. Vaginal delivery, overweight, age, neonatal weight, and weight gain during pregnancy were all risk factors for postpartum urinary incontinence. The vaginal microbiome was no longer *Lactobacillus* dominant of most postpartum women (91.8%), while the diversity of microbiota increased. The *Lactobacillus*-deficient community, commonly labeled as community state type (CST) IV, was sub-divided into four communities. The abundance of vaginal *Lactobacillus* decreased in the group with compromised pelvic muscle functions, while the species richness and diversity increased significantly. In conclusion, the decreased pelvic floor muscle functions of postpartum women 6–8 weeks after delivery may disrupt the balance of vaginal microbiota, and the restoration of pelvic floor functions may contribute to a healthy and balanced vaginal microbiota.

## Introduction

The female pelvic floor is composed of fascia, ligaments, and muscles that maintain the position of pelvic organs (bladder, uterus, vagina, and rectum). Pelvic floor disorders such as pelvic organ prolapse (POP), vaginal laxity, urinary incontinence (UI), and sexual dysfunction may occur when pelvic muscle functions are damaged. The majority of postpartum women will experience pelvic muscle damage to some extent, which is believed to be strongly related to pregnancy and childbirth (Blomquist et al., 2020; Li et al., 2020). The uterus gradually increases during pregnancy, and the uterine and fetal weights before delivery are about 100 times before pregnancy, accompanied by the center of gravity moving forward. Pelvic muscle tissue was loosened under constantly increased pressure. In addition, both the levels of relaxin and the degradation of pelvic floor connective elastin increase during pregnancy, aggravating pelvic floor laxity (Borazjani et al., 2017). Pelvic floor muscles and nerves are extremely stretched during vaginal delivery, especially in the case of obstetric apparatus-assisted vaginal delivery and dystocia, leading to muscle tissue ischemia, degeneration, nerve damage, and even the occurrence of pelvic floor dysfunction.

Previous studies showed that women were more susceptible to vaginal microbiota imbalance 6–8 weeks after childbirth, manifested by a decrease in the proportion and function of *Lactobacillus* and overgrowth of bacterial vaginosis (BV) or aerobic vaginitis (AV) (Doyle et al., 2018). Ravel et al. (2011) summarized five different community state types in women based on different dominant bacterial species. CST I, II, III, and V are dominated by *L. crispatus*, *L. gasseri*, *L. iners*, and *L. jensenii*, respectively, whereas CST IV was characterized by the depletion of *Lactobacillus* spp. A previous study showed that *Lactobacillus* deficiency and increased proportion of CST IV are more common in postpartum women compared to healthy women in the reproductive age (Doyle et al., 2018).

The changes above were considered to be associated with reduced estrogen and glycogen expression in the vaginal epithelium of postpartum women (Hedges et al., 2021). In addition, amniotic fluid and lochia entering the vagina will affect the acidic environment. *Lactobacilli* are heavily outnumbered by their bacterial counterparts, and the microbial balance of vagina is destroyed. On account of decreased pelvic floor muscle strength and vaginal laxity, the vaginal walls may lose tightness, increasing the risk of microbial invasion into internal vagina (Dietz et al., 2018). It may be the underlying mechanism that causes changes in vaginal microbiota, but studies exploring relations between pelvic floor muscle functions and vaginal microbiota are relatively sparse. Therefore, we conducted this study aiming to uncover the relationship between these two conditions.

## Materials and methods

### Participant enrollment

The study was approved by the Biomedical Ethics Committee of Peking University International Hospital, Beijing, China (Ethical Approval No. 2021-KY-0017-02), and followed the STORMS reporting guidelines for human microbiome research (Mirzayi et al., 2021). Postpartum women attending the Department of Obstetrics and Gynecology of Peking University International Hospital for pelvic examination were recruited from December 2021 to April 2022. Written consent and face-to-face questionnaires were obtained from all participants. Vaginal secretion samples were collected from those fulfilling the inclusion criteria, and pelvic floor functions of all the enrolled patients were evaluated. The inclusion criteria were as follows: (a) women who needed pelvic floor function examination 6–8 weeks after delivery; (b) providing signed informed consent form and completing questionnaires. The exclusion criteria were as follows: (a) suffering from puerperal infection or endometritis; (b) vaginal bleeding or sanguinous lochia; (c) using antibiotics or probiotics in the past 30 days; (d) resuming sexual activity after childbirth; (e) vaginal irrigation or drug application performed 7 days before sampling; (f) preexisting hypertension, diabetes, immune system disease, or other infectious diseases such as AIDS, hepatitis, and syphilis. All methods were performed in accordance with the guidelines and regulations.

A total of 230 postpartum women who provided signed informed consent and completed questionnaires were recruited into the trial, and 187 vaginal swab specimens were collected. Four samples were excluded due to failed sequencing reactions, and 183 samples were submitted for analysis. All the 183 postpartum women were divided into two groups defined as postpartum I (PI) group with relatively good pelvic floor functions and postpartum II (PII) group with relatively poor pelvic floor functions to investigate the relationship between pelvic floor functions and vaginal microbiome.

### Pelvic floor function assessment

Pelvic floor muscle strength was assessed by the biofeedback instrument (LABORIE, model Urostym, produced by Laborie Medical Technology, Canada) using pressure assessment (Bø and Sherburn, 2005; Navarro Brazález et al., 2018; Urogynecology Subgroup and Gynecology, 2020). The probe was inserted into the vagina, and participants were asked to perform rapid contractions and endurance contractions of pelvic floor muscles with maximum strength squeezing the probe. Meanwhile, the gluteus and abdominal muscles were not contracted. We recorded the number above 35 peak pressure (in centimeters of water) and duration time (in seconds) with the pressure above 20 as the muscle strength grades of the rapid contractions phase and endurance phase, respectively (Blomquist et al., 2020). Pelvic floor muscle strength was classified into five grades: grade 1 to grade 5. Women scored above grade 3 were categorized into the group with relatively good pelvic floor functions (Friedman et al., 2012).

### Sample collection

Subjects were placed in the lithotomy position on the gynecological examination bed. A well-trained gynecologist used two disposable sterile swabs to collect secretions by swabbing the cervix and vagina 3–5 times and then removed the swab back into the tube. Samples were kept in an ice box and transferred to −80°C storage at the laboratory for subsequent experiment.

### DNA extraction

Fast DNA Spin Kit (MP Biomedicals, Southern California, CA, USA) was used for DNA extraction from cervicovaginal secretions. Each swab was submerged in 2-ml saline. About 200 μl of the samples and 1 ml cell lysis solution (CLS-TC) were added to Lysing Matrix (FastPrep) and then followed the protocol. Eventually, 50 μl liquid containing microbial DNA was obtained. DNA concentration and purity were monitored on 1% agarose gel. According to the concentration, DNA was diluted to 1 g/l using sterile water.

### 16S rRNA gene sequence analysis

V3–V4 hypervariable regions of the 16 S rRNA gene were amplified by PCR using barcoded primers: 341F (5′-CCTAYGGGRBGCASCAG-3′) and 806 R (5′-GGACTACNNGGGTATCTAAT-3′). Mixture PCR products were purified with Qiagen Gel Extraction Kit (Qiagen, Germany). Library preparation was performed following TruSeq^®^ DNA PCR-Free Sample Preparation Kit (Illumina, USA). Qubit@ 2.0 Fluorometer (Thermo Scientific) and Agilent Bioanalyzer 2100 system were used for DNA quality assessment. Finally, the library was sequenced on an Illumina NovaSeq platform, and 250 bp paired-end reads were generated. Paired-end reads were assigned to samples based on their unique barcode and truncated by cutting off the barcode and primer sequence. The reads were merged using FLASH^1^ (V1.2.7) (Magoč and Salzberg, 2011). The raw tags were subjected to specific filtering conditions to obtain high-quality clean tag according to the QIIME^2^ (V1.9.1) quality-controlled process (Caporaso et al., 2010). The tags were compared with the reference database^3^ (Silva database) using UCHIME algorithm^4^ (UCHIME Algorithm) to detect and remove chimera sequences (Haas et al., 2011), resulting in effective tags. Sequence analyses were performed by Uparse software^5^ (Uparse V7.0.1001). Sequences with ≥97% similarity were clustered into the same operational taxonomic units (OTUs). For each representative sequence, the Silva Database (see footnote 3) was used based on Mothur algorithm to annotate taxonomic information (Quast et al., 2013). The unannotated sequences were complemented using BLAST on NCBI nucleotide database (excluding uncultured organisms).

### Statistical analysis

Analysis was executed by SPSS version 22 (IBM, New York, NY, USA) and R software (V4.1.2). Two-tailed test was carried out to find out the statistical difference between groups at 5% significance level. Categorical data were described as frequencies and proportions and compared by Pearson’s chi-squared (χ2) test. Quantitative data were compared by Wilcoxon rank-sum test. Risk factors for weakened pelvic floor functions and urinary incontinence were analyzed by logistic regression calculating the value of odds ratio (OR). By applying analysis of similarities (ANOSIM), analysis of molecular variance (AMOVA), linear discriminant analysis effect size (LEfSe), software (version 1.0), and principal coordinate analysis (PCoA) based on the Bray–Curtis dissimilarity metrics, group differences were uncovered (Excoffier et al., 1992; Schmidt et al., 2018; Hegarty et al., 2020). PCoA was conducted by WGCNA package, stat package, and ggplot2 package in R software, and LEfSe was displayed using the Novomagic, a free online platform for data analysis.^6^ Alpha and beta diversity values were calculated with QIIME software and displayed with R.

## Results

### Pelvic floor functions and influencing factors

To investigate the relationship between pelvic floor functions and vaginal microbiome, we compared the bacterial composition of postpartum I (PI) group and postpartum II (PII) group. Potential factors of pelvic floor muscle function include age, progestational weight, overweight defined as body mass index (BMI) over 24 kg/m2, weight gain during pregnancy, neonatal weight, delivery type, multiparity, postpartum period, and symptom of UI in 6–8 weeks after delivery. Uncontrolled urinary leakage including stress, urge, and mixed urinary incontinence after delivery occurred in 23.0% participants. The incidence of urinary incontinence in group PII was significantly higher than group PI (27.5% vs. 12.5%, *P* = 0.026), but there were no significant differences in remaining factors (Table 1). The percentages of overweight and vaginal delivery were slightly higher in PII group than PI group, but without statistical significance. The detailed information of participants is shown in Supplementary Table 1.

**TABLE 1:** Population characteristics of participants (total *n* = 183).

Variables	PI *n* = 56 (30.6%)	PII *n* = 127 (69.4%)	χ 2/Z	*P*-value
Age (years)	32.04 ± 3.19	31.98 ± 3.51	−0.283	0.777
Progestational weight (kg)	57.30 ± 5.70	57.13 ± 7.22	−0.787	0.431
Overweight (n, %)			2.223	0.136
Yes	6 (10.7%)	25 (19.7%)		
No	50 (89.3%)	102 (80.3%)		
Weight gain during pregnancy (kg)	12.64 ± 3.29	12.57 ± 3.32	−0.248	0.804
Neonatal weight (g)	3257.32 ± 320.43	3269.60 ± 365.25	−0.159	0.874
Delivery mode (n, %)			0.813	0.367
Vaginal delivery	35 (62.5%)	88 (69.3%)		
Cesarean	21 (37.5%)	39 (30.7%)		
Multiparous (n, %)			0.186	0.666
Yes	19 (33.9%)	39 (30.7%)		
No	37 (66.1%)	88 (69.3%)		
Postpartum time (days)	43.84 ± 3.09	43.47 ± 2.76	−1.125	0.261
Urinary incontinence (n, %)			4.984	0.026*
Yes	7 (12.5%)	35 (27.5%)		
No	49 (87.5%)	92 (72.5%)		

PI, postpartum women with relatively good pelvic floor function; PII, postpartum women with relatively poor pelvic floor function; * statistical significance, bold value represents the *P* < 0.05. Quantitative data were shown as mean ± standard deviation compared by Wilcox test, and numeration data were described as percentage compared by Pearson’s chi-squared (χ2) test.

**FIGURE 1:**
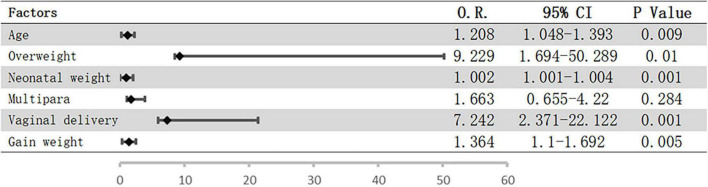
Possible risk factors for UI using logistic regression. Bars represent 95% confidence intervals; O.R., odds ratio; CI, confidence interval; UI, urinary incontinence.

Logistic regression analysis was performed to assess the potential influence of the abovementioned factors on pelvic floor strength. The results showed that only overweight before pregnancy (OR = 4.851; 95% CI = 1.185–19.864; *P* = 0.028) was a risk factor for weakened pelvic floor functions, while the associations with the remaining factors were not significant. However, vaginal delivery and overweight before pregnancy were included when we analyzed the risk factors for urinary incontinence, and the risk of urinary incontinence increased with age, neonatal weight, and weight gain during pregnancy (Figure 1).

### Vaginal microbiota of postpartum women

A total of 16,690 OTUs have been detected in the 183 samples, resulting in a mean sequencing read depth per sample of 85,132 reads (range 56807–96811) utilizing 16S rRNA sequencing. The detailed information of OTUs is shown in Supplementary Table 2.

**FIGURE 2:**
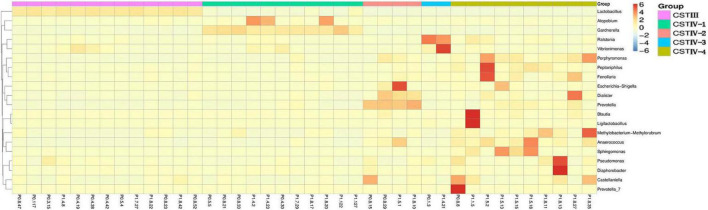
Heat map of the vaginal microbiota with representative 40 participants at genus level. The color key indicates taxon abundance; the order is based on the community state types (CST).

Compared with healthy women of reproductive age in previous studies, the result showed that vaginal microbiome of postpartum women lost *Lactobacillus* dominance, and the bacterial composition was more complex (Figure 2; Ravel et al., 2011; Doyle et al., 2018). The abundance of *Lactobacillus* spp. more than 60% was only 15 (8.2%), and 14 samples were dominated by *L. iners*, while *L. crispatus*, *L. gasseri*, and other species of *Lactobacillus* had no significant dominance. The common anaerobic bacteria *Gardnerella* spp. involved in the pathogenesis of BV showed dominance in six samples (3.3%). The detection rates of *Lactobacillus* species in our samples were listed in the order of abundance as follows: *L. iners* (88.5%), *L. crispatus* (85.2%), *L. gasseri* (68.9%), *L. plantarum* (41.5%), *L. murinus* (32.2%), and *L. jensenii* (24.0%). Surprisingly, *L. jensenii* was detected in only 44 samples in this study, and the relative abundance was very low.

Following the methods of Gajer et al. (2012), we defined the community state types (CSTs) according to the dominance (over 60% of relative abundance in one sample as the threshold). Somewhat unexpectedly, the vaginal microbiome was clustered into two groups in this study, which were resembled CST III dominated by *L. iners* and CST IV. Vaginal L*actobacilli* in most individuals (92.3%) failed to maintain dominance with a more complex bacterial composition, which was classified as CST IV. Not only was CST III in scarcity (7.7%), but CST I, II, and V dominated by *L. crispatus*, *L. gasseri*, and *L. jensenii* were all absent.

**FIGURE 3:**
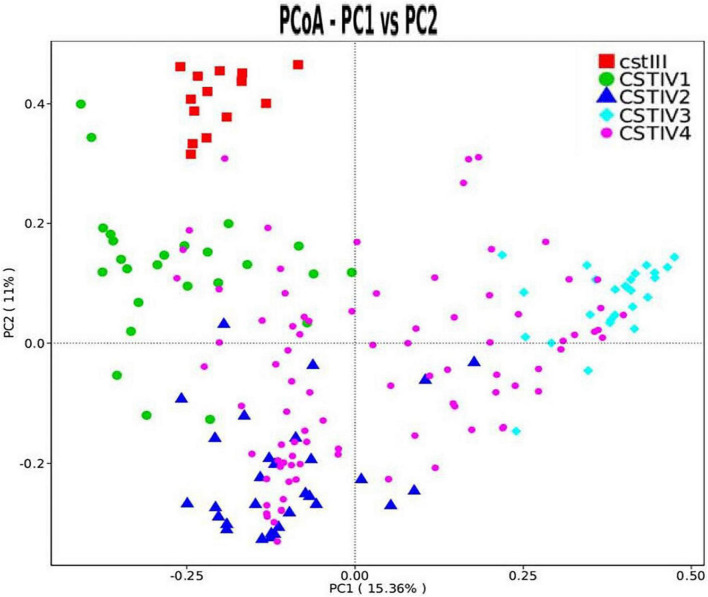
PCoA dissimilarity in CST clusters. Principal coordinates analysis (PCoA) of microbial species data based on Bray–Curtis distance matric displayed the differences of samples between groups. Each dot represented a sample. Different shapes and colors represented corresponding CST.

Our study found that CST IV had obvious subgroups in cluster analysis. Therefore, CST IV was further classified following the method of Doyle et al. However, the microbiota in our study was not dominated by any single OTU but instead showed high abundances of *Gardnerella* spp., *Prevotella* spp., and *Ralstonia* spp. By clustering taxa according to the dominance (over 30% of relative abundance in one sample as the threshold) in genus level, four subgroups of communities in CST IV were defined. CST IV-1, IV-2, and IV-3 were dominated by *Gardnerella*, *Prevotella*, and *Ralstonia*, respectively, while CST IV-4 was not dominated by any genus (Figure 3). Although there was some cross-over in subgroups, these clusters were confirmed as statistically significantly different from each other by AMOVA based on Bray–Curtis dissimilarity metrics (*p* < 0.001).

### The difference of vaginal microbiota between PI and PII group

**FIGURE 4:**
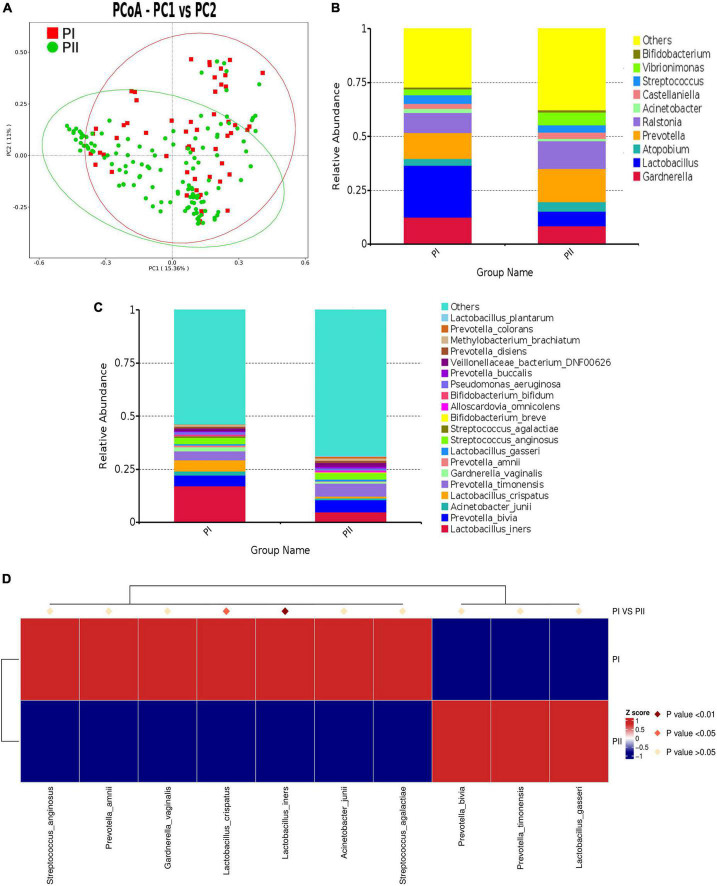
Comparison of microbiota composition between PI and PII Group. **(A)** PCoA dissimilarity between PI and PII. **(B)** Histogram of the relative abundance of vaginal microbiota with top 10 genus in PI and PII groups. **(C)** Histogram of the relative abundance of vaginal microbiota with top 20 species in PI and PII groups. **(D)** Heat map of the vaginal microbiota in PI and PII groups at species level. PI, postpartum women with relatively good pelvic floor function; PII, postpartum women with relatively poor pelvic floor function.

To explore the relations between pelvic floor functions and vaginal microbiota, we made a comparison of the microbiota composition between postpartum women with relatively good pelvic floor functions (PI group) and postpartum women with relatively poor functions (PII group). ANOSIM (*R* value = 0.1171, *P* = 0.001) and AMOVA test (*P* < 0.001) revealed significant differences between two groups which suggested that it was meaningful to classified groups according to pelvic floor function. PCoA was used to cluster community (Figure 4A).

To further investigate the detailed differences between PI and PII group, we compared vaginal microbiota at different levels. We found that, at the phylum level, only the relative abundance of *Bacillota* was significantly higher in group PI (*P* = 0.025), while the abundance of *Bacteroidota* (*P* = 0.001) and *Fusobacteriota* (*P* = 0.01) was significantly higher in group PII. Interestingly, similarity was shown at the class level, as the abundance of *Bacilli* was significantly higher in group PI (*P* = 0.001), while the abundance of *Bacteroidia* (*P* = 0.001), *Fusobacteria* (*P* = 0.011), and *Clostridia* (*P* = 0.001) was significantly higher in group PII (Supplementary Table 3). At genus level, the top four in abundance in PI group were as follows: *Lactobacillus* (24.0%), *Gardnerella* (12.6%), *Prevotella* (12.2%), and *Ralstonia* (9.1%) and in PII group were *Prevotella* (15.4%), *Ralstonia* (12.7) *Gardnerella* (8.5%), and *Lactobacillus* (6.8%) (Figure 4B). The relative abundance for PI and PII group at species level is shown in Figure 4C. Next, we compared the *Lactobacillus* abundance of top six at species level between two groups. The results indicated that *L. iners* was the most abundant in both groups. However, *L. iners* (*Z* = −2.711, *P* = 0.007) and *L. jensenii* (Z = −2.554, *P* = 0.011) were demonstrated to be significantly enriched in PI group (Wilcox test).

To unravel differences comprehensively, we compared microbiota using MetaStat analysis, which revealed significant differences at genus and species levels between two groups (Supplementary Table 4; Figure 4D). To quantify the contribution of each species to the difference between two groups, we used similarity percentage (SIMPER) analysis to decompose the Bray–Curtis difference index, showing the top 10 species in abundance contributing to the differences (Supplementary Figure 1).

**FIGURE 5:**
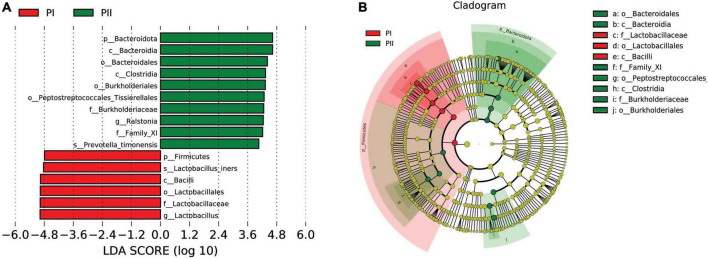
Biomarker between PI and PII. **(A)** Enrichment in microbial taxa with a threshold of LDA score > 4. LDA scores obtained from the LEfSe analysis of the vaginal microbiota in PI and PII groups. LDA, linear discriminant analysis; LEfSe, LDA effect size analysis. **(B)** Cladogram of the vaginal microbiota in PI and PII groups. The microbial compositions were compared at different evolutionary levels.

Linear discriminant analysis effect size (LEfSe) was used to further analyze the difference, uncovering significantly different biomarkers between two groups. As shown in Figure 5, the bacteria in different levels with significant differences and linear discriminant analysis (LDA) score were greater than 4.0, which was the biomarkers with a statistical difference by Kruskal–Wallis rank-sum test (Alpha value = 0.05). We found a significant enrichment of *L. iners* species and *Lactobacillus* with different levels in group PI compared to group PII, while *Bacteroidia* class and *Ralstonia* genus were enriched in PII.

**FIGURE 6:**
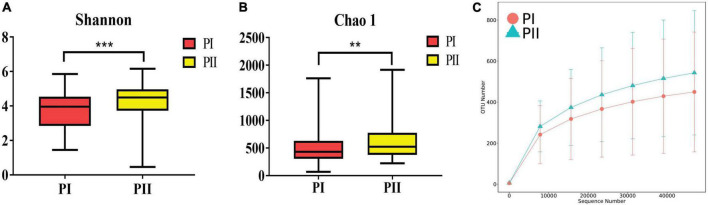
Diversity of the vaginal microbiota between PI and PII. **(A)** Boxplots of the Alpha diversity with Shannon index. **(B)** Boxplots of the Alpha diversity with Chao1 index. **P < 0.01, ***P < 0.001, two-sided Wilcoxon test. Bounds of boxes showed interquartile range (IQR) with the lower and upper hinges corresponding to the 25th and 75th percentiles, respectively. **(C)** Rarefaction curve showed the OTU numbers increased more with enlargement of sequence numbers in PII group.

Alpha diversity was measured using the Shannon, Chao1, ACE, and Simpson indexes to determine whether alpha diversity differed between groups. The four diversity indexes showed consistent results that the community richness and diversity of group PII were significantly higher than group PI (*P* < 0.01) (Figure 6). Similarly, a significant difference in β diversity was also observed (Wilcox, *P* = 0.006) based on the binary Jaccard test in two groups.

### Comparison of vaginal microbiota between urinary incontinence group and no urinary incontinence group

The subjects were divided into urinary incontinence (UI) group and no urinary incontinence (NUI) group to compare the composition of vaginal microbiota. The results showed that the relative abundance of *Gardnerella* in UI group was considerably higher than that in NUI group, but the results were not statistically significant (*p* = 0.14). There were no significant differences in *Lactobacillus* abundance, alpha diversity index, PCoA, ANOSIM test, and AMOVA test between two groups (Supplementary Figures 2A,B). However, we found that *Acinetobacter* (*Z* = −2.76, *p* = 0.006) genus was significantly abundant in NUI group, while *Castellaniella* (z = −2.10, *p* = 0.036) was significantly abundant in UI group, despite the low relative abundance of them in vaginal. LEfSe analysis showed similar results (Supplementary Figures 2C,D).

## Discussion

Previous studies showed that the incidences of abnormal pelvic floor functions and UI in postpartum women were (50–64%) and (23–32%), respectively, which were basically consistent with our results (Qi et al., 2019; Yang et al., 2019; Patel et al., 2021). In this study, we concluded that more than half of (69.4%) postpartum women 6–8 weeks after delivery had abnormal pelvic floor functions, while the incidence of urinary incontinence UI was sharply lower (23.0%). Logistics analysis showed that overweight before pregnancy was a risk factor for pelvic floor dysfunction. However, there were more risk factors for postpartum women with urinary incontinence. Considering that urinary incontinence is a symptom of severe pelvic floor dysfunction, the correlation of risk factors is more pronounced than symptomless women, but researches have so far proved inconclusive results about which factors (age, weight, delivery type, and fetal weight) wielded more influences on pelvic floor functions and the occurrence of UI (Qi et al., 2019; Blomquist et al., 2020; Li et al., 2020; Fu et al., 2021; Patel et al., 2021).

Previous studies reported that vaginal microbiota during pregnancy is less diverse and more stable than postpartum period, which determined that CST types in postpartum women were different from non-pregnant women (Macintyre et al., 2015; Zhang et al., 2022). It was also demonstrated in the research of Doyle et al. (2018) despite a few differences. The similarities included that only two clusters labeled CST III and CST IV were obtained in two studies, and the majority of participants had a *Lactobacillus* spp. deficient community that was replaced by microbiota similar to CST IV in previous studies. A longitudinal study indicated that the vaginal microbiome lacked a certain proportion of *Lactobacillus* after delivery, while the bacterial diversity increased. The most prevalent CST observed in healthy Chinese pregnant women was CST I, while CST IV was more observed in the postpartum samples (Zhang et al., 2022). Another longitudinal study focused on European populations reported similar results that CST IV had a significant increase, approximately 75% in all postpartum samples (Macintyre et al., 2015). An American survey showed that around 77% of the postpartum vaginal communities had a diverse array of bacteria, including *G. vaginalis*, *Prevotella* spp., *Streptococcus* spp., and numerous others, with low proportions of *Lactobacillus* spp. (Nunn et al., 2021). Similar results were observed in our study. Except for *L. iners* the microbiota was not dominated by any single OTU or specie but instead with relative medium abundances of *Gardnerella* spp., *Prevotella* spp., and *Ralstonia* spp. resembling CST 4-III in the reports of Doyle et al. (2018). In conclusion, the dominant species of CST IV varied in different studies, but CST IV was the dominant type in postpartum vaginal secretions. Those studies shared common results of reduced *Lactobacillus*, complex microbiota, and increased microbiome diversity in postpartum samples.

Most of the previous studies have focused on the impacts of hormonal changes, age, delivery mode, geographical distribution, postpartum time, and HIV infection on vaginal microenvironment (Curty et al., 2017; Balle et al., 2020). Nunn et al. (2021) considered that the decrease in relative abundance of *Lactobacillus* in postpartum women might be associated with the maternal estrogen levels falling precipitously after placenta removal. Selma-Royo et al. (2020) found that the mode of delivery influenced vaginal microbiota composition manifesting the changes of relative abundance of various genera. Therefore, it is worthwhile further exploring factors contributing to the changes of vaginal microbiota, especially in postpartum women. In this study, we classified participants into two groups according to pelvic floor functions to explore the influence of pelvic floor functions on vaginal microbiota. Both ANOSIM and AMOVA analysis showed significant differences in vaginal microbiota composition between two groups.

The reasons for the differences are worth pondering. Unfortunately, we have not found any report about the association between these two conditions especially using 16S rRNA gene sequencing. It was considered that the unsatisfactory results of pelvic floor muscle pressure assessment were closely related to the weakness of muscles and the looseness of vaginal wall or vaginal laxity. Pressure from the growing uterus during pregnancy, decreased collagen fibers, and damaged myofibrils had adverse effects on pelvic floor muscle functions. Especially during vaginal delivery, the vaginal walls and introitus were overstretched, and levator ani muscle (LAM) was subjected to extensive stretch even tear, weakening the support for pelvic organs support and leading to vaginal laxity (Manzini et al., 2020; Fu et al., 2021; Van Gruting et al., 2021). The vagina was unable to maintain closed, and the mucosal folds became flattened because of excessive extension, leading to weakened mucosal barrier. In consequence, internal vaginal environment was susceptible to disruption. In severe cases, recurrent vaginitis occurred and the adjacent lower urinary tract was contaminated, causing inflammation of the urinary system, overactive bladder, and even urinary leakage.

Komesu et al. (2020) reported that there was a significant correlation between vaginal and urinary microbiota, both of which were dominated by *Lactobacillus* and interacted with each other to influence urinary and reproductive health. Studies revealed that intravaginal instillation of *Lactobacilli* might prevent recurrent urinary tract infections and the increased abundance of *Lactobacillus* in urinary tract and bladder was associated with the relieving overactive bladder symptoms (Stapleton et al., 2011; Pearce et al., 2014; Thomas-White et al., 2020). In our study, postpartum vaginal microbiota was not significantly correlated with urinary incontinence, considering the fact that subjects in our trial were all postpartum women and the number of UI participants in our experiment was relatively small. Another possible factor was that changes in vaginal microbiota were more closely associated with vaginal laxity, a manifestation of levator ani hyperdistensibility, compared to urinary incontinence caused by anatomical abnormalities of bladder and urethra. These results suggested it was essential to further explore the relationship between vaginal microbiota, pelvic floor muscle function, and urinary symptoms.

Several potential factors that may influence the composition of the vaginal microbiome have been explored, but this is the first study to describe the relationship between vaginal microbiome and pelvic floor function using 16S rRNA sequencing. The study revealed that anatomical changes such as impaired pelvic floor muscles and vaginal laxity were risk factors of vaginal microbiota disorder. Combining with the high incidence of pelvic floor dysfunction and the low *Lactobacillus* levels in elderly women, the decline of pelvic floor function may also play a significant role in addition to the influence of decreased hormone level (Brotman et al., 2014; Dumoulin et al., 2019; Geng et al., 2020). The study highlighted the importance of postpartum pelvic floor exercises in other perspective, and the outcome might shed light on the treatment of postpartum and senile vaginitis with pelvic floor exercises.

This study showed changes in microbiota as pelvic floor functions declined. However, we do not have Nugent criteria and symptoms of vaginitis results to evaluate associations with pelvic floor function. For future research, the samples for wet mount using phase-contrast microscopy to evaluate the status of AV and BV are needed to understand the relationship of pelvic floor muscle function and different vaginitis. To verify the reliability of the conclusions, further investigations with expanded population like elderly women, not only postpartum women, are essential to evaluate the comprehensive relationship between pelvic floor muscle function, vaginal relaxation, and vaginal microbiota. Regardless, insights gained from the trail may contribute to the potential causes of vaginal complexity microbiota and improved pelvic floor function may be beneficial to women vaginal health.

## Data availability statement

The datasets presented in this study can be found in online repositories. The names of the repository/repositories and accession number(s) can be found in the article/Supplementary material.

## Ethics statement

The studies involving human participants were reviewed and approved by Biomedical Ethics Committee of Peking University International Hospital, Beijing, China. The patients/participants provided their written informed consent to participate in this study.

## Author contributions

GG and YZ designed the study. GG, LL, and GX directed and coordinated all aspects of this study. YZ and WY performed acquisition of samples and data. YZ and HY contributed to the laboratory work, data analysis, and plotting pictures. YZ wrote the original manuscript. All authors contributed to review and revision of the manuscript.

## Footnotes

1
http://ccb.jhu.edu/software/FLASH/

2
http://qiime.org/scripts/split_libraries_fastq.html

3
https://www.arb-silva.de/

4
http://www.drive5.com/usearch/manual/uchime_algo.html

5
http://drive5.com/uparse/

6
https://magic.novogene.com
