# Supplementary material for: The relationship between pelvic floor functions and vaginal microbiota in 6–8 weeks postpartum women
Source: Front Microbiol. 2022 Nov 3;13:975406. doi: 10.3389/fmicb.2022.975406 (PMC9669797; doi:10.3389/fmicb.2022.975406)
Supplement: Supplementary file 1 [file Data_Sheet_1.PDF]

Supplementary Material

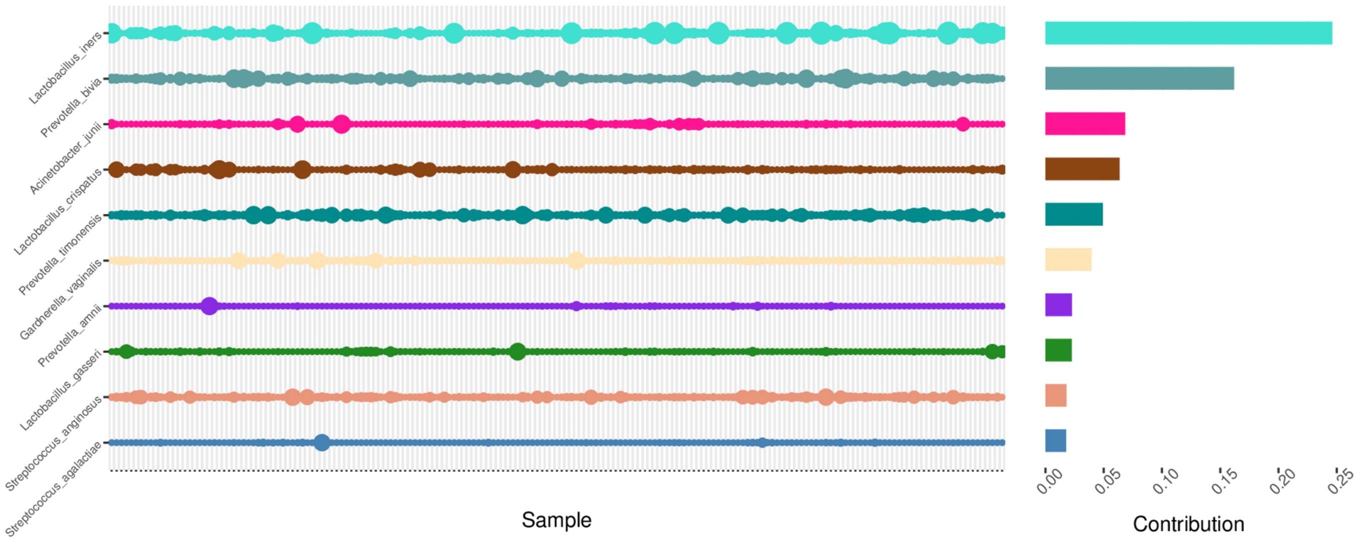

Supplementary Figure 1. Simper analysis based on the Bray-Curtis difference with the top 10 species contributing to the difference between PI and PII
The size of the dot represents the relative abundance of species of the sample.

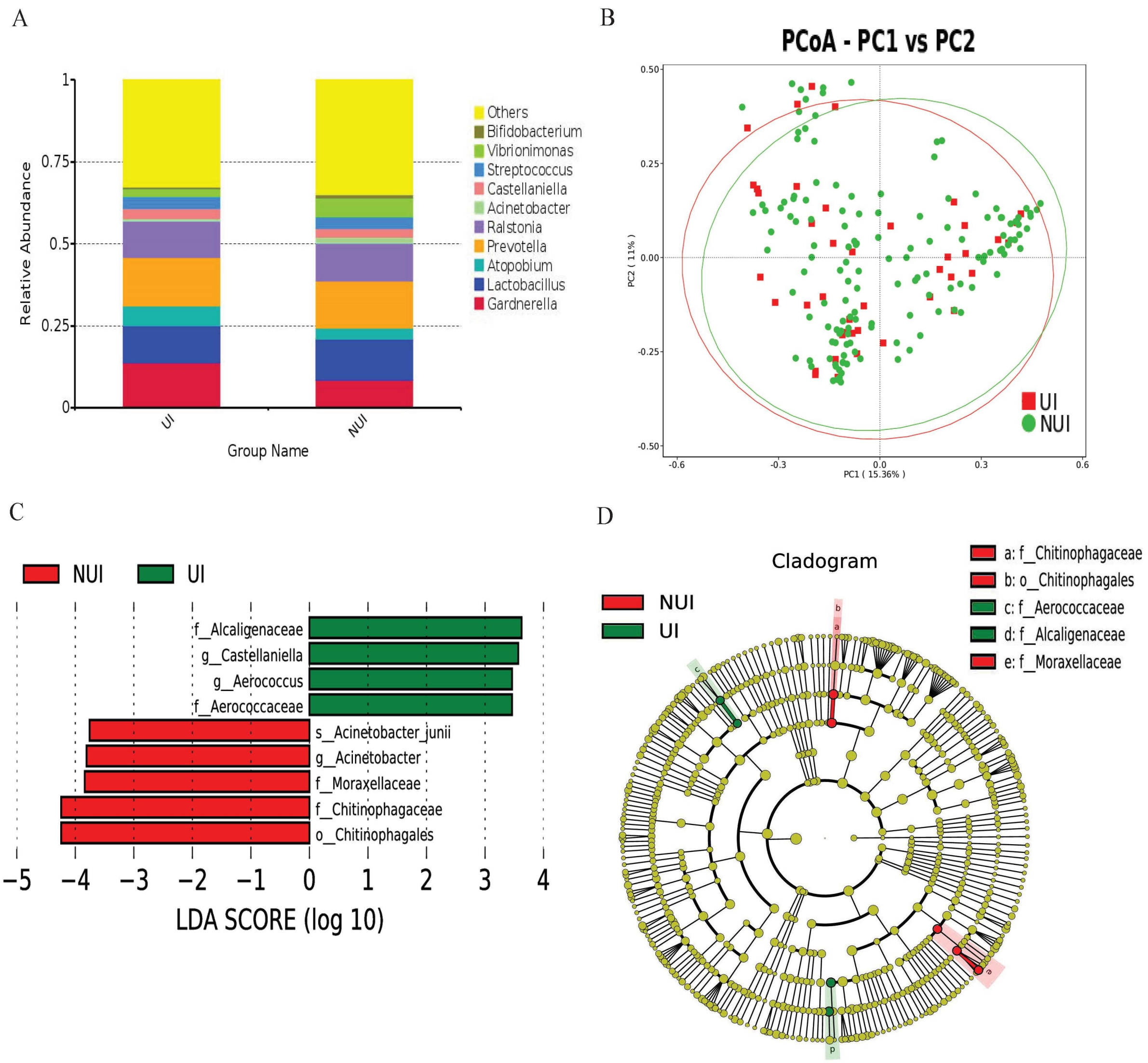

Supplementary Figure 2. The comparison of microbiota composition between UI and NUI group (A). Histogram of the relative abundance for groups at genus level. (B). PCoA dissimilarity between groups. (C) Enrichment in microbial taxa with a threshold of LDA score > 3. LDA scores obtained from the LEfSe analysis of the vaginal microbiota in UI and NUI groups. LDA, linear discriminant analysis; LEfSe, LDA effect size analysis. (D) Cladogram of the vaginal microbiota in UI and NUI groups. The microbial compositions were compared at different evolutionary levels. UI: urinary incontinence group; NUI: no urinary incontinence group

Supplementary Table 1. The detailed population characteristics of all participants (total n=183)

Supplementary Table 2. The detailed OTUs information of all participants

Supplementary Table 3. Relative abundance for all participants at class level

Supplementary Table 4. MetaStat analysis for PI and PII group at genus and species levels
